# Characterization of the effects of oxytocin on fear recognition in patients with schizophrenia and in healthy controls

**DOI:** 10.3389/fnins.2013.00127

**Published:** 2013-07-18

**Authors:** Meytal Fischer-Shofty, Simone G. Shamay-Tsoory, Yechiel Levkovitz

**Affiliations:** ^1^Department of Psychology, University of HaifaHaifa, Israel; ^2^Shalvata Mental Health Care CenterHod Hasharon, Israel

**Keywords:** oxytocin, schizophrenia, emotions, fear, emotion recognition

## Abstract

Individuals who suffer from schizophrenia often show a marked deficit in recognition of emotional facial expressions, as part of broader impairment of social cognition. Research has shown that recognition of negative emotions, specifically fear recognition, is particularly impaired among patients with schizophrenia. Recently we reported that intranasal administration of OT (IN OT) increased the ability to correctly recognize fear in a group of healthy men. The aim of the current study was to examine the effects of IN OT administration on fear recognition among patients with schizophrenia. Based on previous research, we also sought to examine a possible selective effect of OT dependent on baseline performance, hypothesizing that IN OT would have a greater enhancement effect on less proficient individuals. It was thus hypothesized that patients will show more improvement in fear recognition following the administration of IN OT as compared to controls. Sixty six participants (31 schizophrenia patients, 35 healthy controls) were enrolled in the current study. All participants received treatment of a single dose of 24 IU IN OT and an equivalent amount of placebo, 1 week apart. The participants' ability to accurately recognize fear and happiness was evaluated using a face morphing task. Overall, as a group, both patients and healthy control participants were more accurate in recognizing fearful facial expressions, but not happy faces, following IN OT administration, as compared to their performance following placebo. IN OT did not differentially affect emotion recognition in patients and healthy controls. Yet, the results indicated a selective effect for IN OT, in which the hormone improves fear recognition only among individuals whose baseline performance was below the median, regardless of their psychiatric status.

## Introduction

Individuals who suffer from schizophrenia often show a marked and stable deficit in many aspects of social cognition, such as emotion recognition, social perception, and empathy (Brune, 2005; Green et al., 2005, 2012; Derntl et al., 2009; Kohler et al., 2010; Achim et al., 2011). These deficient abilities are consistent over time, apparent in the prodrome to psychosis (Amminger et al., 2012a,b; Thompson et al., 2011, 2012) and seriously impede social competence (Brune et al., 2007; Smith et al., 2012). Indeed, they have a tremendous impact on overall proficiency and rehabilitation results, leading Wible (2012) to define this illness as one of “social communication.” Furthermore, social cognition has been suggested as a mediator between neurocognition and functional outcome, demonstrating once more this domain's prominent role in this disease (Green et al., 2012).

Numerous studies of schizophrenic patients over the last decades have shown a consistent and marked impairment in emotion recognition via facial expression (Pinkham et al., 2007; Kohler et al., 2010), prosody (Edwards et al., 2001, 2002; Hoekert et al., 2007; Roux et al., 2010; Jahshan et al., 2013), and whole body movements (Singh et al., 2011). Furthermore, impairments in emotion recognition have been shown to predict functional outcome for schizophrenic patients (Irani et al., 2012).

Facial emotion recognition has been widely investigated in the context of schizophrenia, due to its correlation with global functioning, as well as the fact that patients with schizophrenia show a consistent impairment in this domain. Furthermore, previous studies have suggested that compared with the recognition of other emotions (i.e., happiness), patients with schizophrenia may have a selective dysfunction in the perception of negative emotions such as fear (Kohler et al., 2003; Hofer et al., 2009; Huang et al., 2011; Amminger et al., 2012a,b; Chen et al., 2012). For example, Chen et al. (2012) reported a deficit in fear discrimination in patients with schizophrenia compared to healthy control subjects, as well as a significant correlation between fear discrimination and *Positive and Negative Symptoms of Schizophrenia* (PANSS) negative symptoms scores. A specific impairment in fear recognition was also evident in a group of patients with schizophrenia (Leung et al., 2011), as well as in the studies of Norton et al. (2009) and Amminger et al. (2012a,b). These findings suggest that while fear recognition may not encompass the whole social cognition deficit shown by patients with schizophrenia, it might have a considerable importance with respect to this disorder. Impaired fear recognition has been associated with maladaptive aggressive behavior in schizophrenia (Weiss et al., 2007), as well as in other socially impaired populations (Marsh and Blair, 2008). Earlier, Marsh et al. (2007) showed evidence to support that fear recognition in healthy participants is strongly associated with prosocial behavior. Thus, it is possible that improving fear recognition in schizophrenia may be valuable in diminishing their levels of social impairment and improve their daily interactions.

We have recently developed a novel explanatory model of social impairments in schizophrenia that focuses on dysfunction in the oxytocinergic system (Fischer-Shofty et al., 2013). Oxytocin (OT) has repeatedly been shown to improve different facets of social cognition, such as emotion recognition (Fischer-Shofty et al., 2010; Marsh et al., 2010; Lischke et al., 2012a,b; Van and Bakermans-Kranenburg, 2012), empathy (Domes et al., 2007a,b; Bartz et al., 2010; Krueger et al., 2013), and trust (Kosfeld et al., 2005; Theodoridou et al., 2009). Recently, Lischke et al. (2012a,b) reported that intranasal administration of OT (IN OT) increased participants' ability to detect emotion in facial expressions, as compared to a placebo group. Schulze et al. (2011) demonstrated a similar effect of enhanced emotion detection following IN OT administration, as expressed in more accurate performance on tasks involving emotion recognition of masked facial expressions. Consistent with these studies, we previously reported that intranasal administration of OT specifically improved accurate recognition of fearful facial expressions (Fischer-Shofty et al., 2010). An improvement in emotion recognition, and that of fear in particular, following OT administration was also evident in the recent meta-analysis of Shahrestani et al. (2013), adding further evidence for the possible role of OT in fear recognition.

Thus, the oxytocinergic system is a promising neuromodulator of emotion recognition that may have the potential to normalize the social dysfunction seen in schizophrenia. Indeed, studies have shown abnormal levels of OT in the plasma of patients with schizophrenia (Goldman et al., 2008), as well as in their cerebrospinal fluid (CSF) (Linkowski et al., 1984; Beckmann et al., 1985; Legros et al., 1992). Moreover, OT levels have been significantly associated with social functioning of patients (Rubin et al., 2010; Sasayama et al., 2012), and recent studies have linked variations in the OXTR gene to schizophrenia (Souza et al., 2010a,b; Montag et al., 2012a,b). In addition, previous studies suggest an antipsychotic influence of OT among individuals who suffer from schizophrenia (Bujanow, 1974; Feifel and Reza, 1999; Caldwell et al., 2009; Feifel et al., 2010; Pedersen et al., 2011; Macdonald and Feifel, 2012). Recently Feifel et al. (2012) reported a beneficial effect for IN OT on verbal memory in schizophrenia. With respect to social cognition, OT was reported to improve emotion recognition among schizophrenic patients (Averbeck et al., 2012), as well as theory of mind and social judgments (Pedersen et al., 2011).

In view of the role of OT in facial emotion recognition (especially fear recognition), as well as the established deficit of patients with schizophrenia in emotion recognition, we sought to examine the effect of IN OT on fear recognition in individuals with schizophrenia. In light of our previous research (Fischer-Shofty et al., 2010) demonstrating that a single dose of IN OT significantly improved fear recognition among healthy men, we hypothesized that patients with schizophrenia would show improved fear (but not happiness) recognition following the administration of IN OT. Second, we sought to compare the effect of IN OT on emotion recognition in patients to that of healthy control individuals. Moreover, based on earlier results exhibiting a selective effect of IN OT on less socially competent individuals (Bartz et al., 2010), we hypothesized that the effect of IN OT administration on fear recognition would be stronger among patients compared to among healthy controls.

## Methods

### Participants

Thirty patients diagnosed with schizophrenia (27 men and 3 women, mean age = 31.8 years, *SD* = 6.53) and thirty-five age-matched healthy individuals (32 men and 3 women, mean age = 29.49 years, *SD* = 5.59) participated in the current experiment, all of them were native Hebrew speaking. Two senior psychiatrists diagnosed the patients according to the DSM-IV criteria by means of structured clinical interviews. In addition, all of the participants were screened by a trained clinician for various psychopathologies using the Mini International Neuropsychiatric Interview (M.I.N.I.) (Sheehan et al., 1998). Exclusion criteria were physical illnesses (including arrhythmia, psychiatric conditions, any neurological condition, and head injury); IQ below 75; disturbances in visuomotor coordination; and alcohol or drug abuse. The participants were outpatients and day-hospital patients receiving stable doses of relevant medication (first generation and second generation antipsychotics, benzodiazepines, anticholinergic drugs). All of them gave their oral and signed consent. All participants were instructed to avoid using psychotropic substances (e.g., caffeine and nicotine) for at least 12 h prior to the experiment. The study was approved by Israel's National Institutional Review Board as well as by the Helsinki committee of the Shalvata Mental Health Center, and was registered as a clinical trial at the NIH website (code number NCT00813436). Patients and controls were age matched, and all received financial compensation for participating in the study.

### Clinical assessment

As shown in Table 1, the patients were clinically assessed by a trained psychologist using the PANSS, developed by Kay et al. (1987) to evaluate positive symptoms, negative symptoms and general psychopathology (occurrence and severity), and the *Clinical Global Impression scale* (CGI), used to estimate the severity of patients' illness. All of the participants completed the vocabulary subtest and the abstract test of the *Shipley Institute of Living Scale* (Shipley, 1940) to assess their intellectual abilities. For technical reasons, only 42 subjects overall completed the Shipley scale.

**Table 1:** **Demographic and clinical characteristics of patients with schizophrenia and controls**.

	**Patients with schizophrenia (*n* = 31)**	**Control participants (*n* = 35)**	**Statistics**	***p***
Age (years)	31.80 (6.53)	29.49 (5.59)	*t*_(63)_ = −1.541	0.128
Gender (males: females)	27:3	32:3		
Education (years)	12.20 (1.97)	15.09 (2.02)	*t*_(63)_ = 5.804	0.000
Illness duration (years)	11.02 (6.73)			
Number of hospitalizations	3.48 (3.34)			
PANSS total score	68.55 (12.41)			
PANSS positive symptoms	15.59 (3.92)			
PANSS negative symptoms	18.48 (4.56)			
PANSS general psychopathology	34.10 (6.59)			
CGI	4.63 (0.81)			
Shipley vocabulary	25.73 (7.47)	30.50 (6.91)	*t*_(40)_ = 1.906	0.064
Shipley abstract	10.73 (4.33)	14.00 (3.30)	*t*_(40)_ = 2.637	0.024

### Treatment administration

In the current study we employed a double-blind within-subject crossover design. All participants participated in two sessions, one after IN OT administration and the other 7 days later, following placebo administration. Half of the participants were randomly assigned to receive IN OT in the first session, and half began with placebo administration. In both sessions the behavioral tasks began 45 min after substance administration. IN OT administration included three puffs of syntocinon spray (Novartis) in each nostril (each puff containing 4 IU, a total of 24 IU). Placebo administration involved three puffs in each nostril from a similar looking spray bottle that contained all the inactive ingredients except for OT. IN OT dosage and waiting time corresponded to those used in previous experiments investigating the effect of intranasal administration of OT on human behavior (Kirsch et al., 2005; Kosfeld et al., 2005; Domes et al., 2007a,b, 2010; Guastella et al., 2008).

### Assessment of facial emotion expression recognition: the facemorphing task

The facemorphing task was designed to test recognition of emotional facial expressions. In the task, gradually changing facial expressions are portrayed, beginning with a neutral expression that continuously evolves into an emotional expression. The faces were gray-scale standardized computer-generated photographs of six Caucasian participants, whose facial expressions varied to express happiness or fear. The face stimuli included the eye and nose regions, while the mouth region was masked. The participant's task was to correctly identify the emotional facial expression as soon as possible. The facemorphing task was used in view of its advantage in mimicking true emotional facial expressions, which gradually appears in the context of interpersonal interaction.

The face stimuli were images of three men and three women from the Ekman series (Ekman and Friesen, 1976). Each face stimulus was positioned within a rectangular frame measuring 6.1 × 8.9 cm, subtending 5.0 × 7.3° of visual angle at a 70-cm viewing distance (173 × 251 pixels on a 256 gray-level scale). We used graphic image morph software (Face Morph Lite 2.0) to generate gradually evolving emotional facial expressions, from neutral to emotional expression. The emotional facial expression recognition task included twelve stimuli (six stimuli of each emotion, e.g., fear and happiness), generated by e-prime 2.0 software. The stimuli were presented at a frame ratio of 10 fps (each frame was presented for 100 ms) and included 100 frames, for a total of 10 s. In the task, participants were asked to press the spacebar key as soon as they recognized an emotion in the gradually changing expression. Immediately after pressing the spacebar key, they were asked to report which emotion they recognized from a list of six basic emotions (happiness, sadness, anger, fear, disgust, and surprise). Reaction time and frame onset were recorded as dependent variables.

### Assessment of mood

In the current study we used an adapted version of the Depression Adjective Check Lists (DACL; Lubin, 1965) to evaluate participants' general mood following administration of each of the substances (IN OT and placebo). The DACL is a self-report instrument that includes a list of 32 adjectives describing various mood states. Participants were asked to choose the words on the list that best describe their current mood. We calculated the number of positive and negative adjectives each participant chose during each session.

### Statistical analyses

Analyses were conducted using SPSS (version 17). To assess treatment effect in patients and in controls, repeated measures ANOVA was used. Follow-up *t*-tests (with Bonferonni corrections) were conducted in order to further explore simple effects. Moreover, a regression model was used in order to examine the possible relationship between baseline performance level of fear recognition and the amount of improvement following IN OT administration. Once again, follow-up *t*-tests were conducted in order to further examine significant effects. Bonferonni corrections were used to control for multiple comparisons, for both the repeated measure ANOVA and the regression model.

## Results

Independent-sample *t*-tests were employed to identify the demographic and clinical characteristics of both groups, as well as to evaluate group differences (see Table 1).

### General effects of IN OT on mood

A 2 × 2 × 2 repeated measures ANOVA indicated no overall treatment effect [*F*_(1, 60)_ = 0.038, *p* = 0.846] or interaction of mood type (sadness, happiness, lack of energy, depressed, calm, etc.) by treatment [*F*_(7, 54)_ = 1.864, *p* = 0.094] for mood ratings. The three-way interaction (treatment, mood type, group) was not significant, [*F*_(7, 54)_ = 1.597, *p* = 0.156], further confirming that IN OT did not have a differential effect on the mood of patients and controls. There was a general group effect [*F*_(1, 60)_ = 7.331, *p* = 0.009], demonstrating an overall difference in mood ratings between healthy controls and patients. Although patients [Mean = 4.49 (*SD* = 1.22)] and control participants [Mean = 4.77 (*SD* = 0.96)] did not differ on their positive mood ratings, patients with schizophrenia had higher ratings of negative mood [Mean = 2.65 (*SD* = 1.22)] as compared to controls [Mean = 1.83 (*SD* = 0.81)].

### IN OT and emotional facial expression recognition

**Figure 1:**
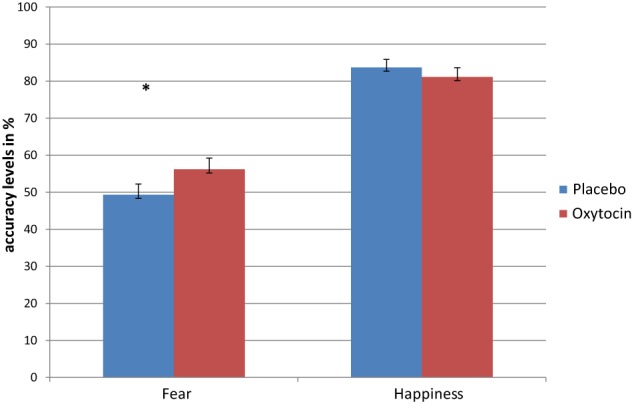
**Repeated measures analysis shows a significant interaction between treatment (OT/placebo) and emotional facial expression (fear/happiness), indicating a differential effect of OT and placebo treatments on recognition of fearful facial expressions, as opposed to happy expressions for all participants**. Separate repeated ANOVA indicated significant treatment effect only for fear recognition (^*^), but not for the recognition of happiness (^*^p < 0.05).

To examine the interaction among treatment, emotional facial expression, treatment order and group membership, a four-way (2 × 2 × 2 × 2) repeated measures ANOVA (with Bonferonni corrections) was conducted, with type of treatment administered (IN OT, placebo) and emotional facial expression (fearful, happy) as within-subjects factors and treatment order (IN OT first, placebo first) and group membership (patients, controls) as between-subjects factors. As shown in Figure 1, a significant emotional facial expression effect was evident [*F*_(1, 61)_ = 164.88, *p* = 0.0001], partial η^2^ = 0.730, indicating that participants across groups were better at recognizing happiness [Mean = 82.4 (*SD* = 0.018)] than fear [Mean = 52.73 (*SD* = 0.026)]. In addition, a significant group effect was found [*F*_(1, 61)_ = 6.251, *p* = 0.015], partial η^2^ = 0.102, indicating that patients scored lower on the task [Mean = 62.32 (*SD* = 0.032)] as compared to controls [Mean = 72.06 (*SD* = 0.0205)]. Moreover, a significant interaction between treatment and emotional facial expression was found [*F*_(1, 61)_ = 5.091, *p* = 0.028], partial η^2^ = 0.077, indicating a differential effect of IN OT and placebo treatments on recognition of fearful facial expressions as opposed to happy expressions for all participants.

To further examine the significant interaction between treatment and emotional facial expression, we conducted follow-up paired samples *t*-tests, which indicated that while there was no significant difference [*t*_(64)_ = −0.88 ns] between accuracy levels for recognition of happy facial expressions following IN OT administration [Mean = 81.11 (*SD* = 20.45)], as compared to following placebo administration [Mean = 83.69 (*SD* = 17.64)], a significant difference [*t*_(64)_ = 2.35, *p* = 0.022] was evident for recognition of fearful facial expressions. The accuracy level for recognition of fear following IN OT administration [Mean = 56.17 (*SD* = 24.65)] was significantly higher than accuracy levels following placebo administration [Mean = 49.29 (*SD* = 23.59)], Cohen's *d* = 0.292.

Moreover, the ANOVA described before did not reveal any significant treatment effect [*F*_(1, 61)_ = 1.238, *p* = 0.270], indicating no general difference in emotion recognition following IN OT administration as compared to placebo administration. No significant interactions were found between treatment and group membership (schizophrenic patients, healthy control) [*F*_(1, 61)_ = 0.849, *p* = 0.360], treatment and order of treatment administration [*F*_(1, 61)_ = 0.156, *p* = 0.694], emotional facial expression and group membership [*F*_(1, 61)_ = 1.915, *p* = 0.171] or emotional facial expression and order of treatment administration [*F*_(1, 61)_ = 0.709, *p* = 0.403]. In addition, the three-way interaction of treatment × emotional facial expression × treatment order was not significant [*F*_(1, 91)_ = 1.966, *p* = 0.166], nor was the three-way interaction of treatment × emotional facial expression × group membership [*F*_(1, 61)_ = 0.529, *p* = 0.470]. Finally, the four-way treatment × emotional facial expression × group membership × treatment was also not significant [*F*_(1, 61)_ = 0.112, *p* = 0.739].

In order to check whether our non-significant interaction results were due to a lack of statistical power, we conducted *post-hoc* power analyses using GPower (Erdfelder et al., 1996) with power (1 − β) set at 0.80 and α = 0.05, two-tailed. This showed us that total sample sizes would have to increase up to *N* = 168, in order the interaction effect to reach statistical significance at the 0.05 level.

In addition, we reanalyzed the data without female subjects (3 from the patients' group and 3 from the healthy control group, 6 overall). The four-way (2 × 2 × 2 × 2) repeated measures ANOVA indicated that although the main effects of emotional facial expression [*F*_(1, 55)_ = 155.06, *p* = 0.0001], partial η^2^ = 0.738, and group [*F*_(1, 55)_ = 5.19, *p* = 0.027], partial η^2^ = 0.086, were significant, the interaction between treatment and emotional facial expression was only marginally-significant [*F*_(1, 55)_ = 3.93, *p* = 0.052], partial η^2^ = 0.067.

To confirm that intellectual abilities did not affect the performance in the task, we conducted a repeated measures ANCOVA, using both years of education and intelligence (Shipley score) as covariates in the whole sample, as well as using treatment order (IN OT first, placebo first) and group membership (patients, controls) as between-subjects factors. This analysis indicated that the interaction between treatment and emotional facial expression remains significant [*F*_(1, 36)_ = 6.44, *p* = 0.016], partial η^2^ = 0.152, suggesting that the years of education and intelligence level as measures by the Shipley test do not affect the IN OT × emotional facial expression recognition interaction, but rather improves it.

In regard to reaction time, we conducted a four-way (2 × 2 × 2 × 2) repeated measures ANOVA to examine the relationship between treatment, emotional facial expression, treatment order, and group membership. A significant emotional facial expression effect was evident [*F*_(1, 52)_ = 64.14, *p* < 0.0001], partial η^2^ = 0.552, indicating that subjects' reaction time while facing fear [Mean = 2089.1 (*SD* = 507.59)] was higher than while recognizing happiness [Mean = 1537.25 (*SD* = 669.66)]. In addition, a significant group effect was evident [*F*_(1, 52)_ = 14.625, *p* < 0.0001], partial η^2^ = 0.220, indicating that patients' reaction time overall was higher [Mean = 2083.11 (*SD* = 460.32)] as opposed to healthy control group [Mean = 1581.81 (*SD* = 413.08)]. No interaction between this two factors was evident [*F*_(1, 52)_ = 0.586 ns].

**Figure 2:**
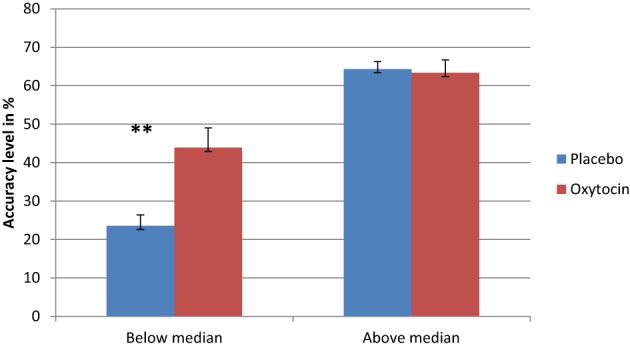
Separate paired *t*-tests reveal that participants whose basic fear recognition ability was below the median were significantly improved in fear recognition following OT administration, as opposed to their performance following placebo administration (^**^). Conversely, participants whose basic fear recognition ability was above the median did not exhibit any significant difference between their OT performance and placebo performance (^**^p < 0.0001).

As in previous studies that found a differential drug effect among participants with lower performance (Kimberg et al., 1997; Mattay et al., 2000; Farah et al., 2009), we sought to examine the possibility that IN OT has a differential effect on individuals, depending on their baseline abilities. To examine this possible effect, we conducted a regression analysis using the difference in performance between IN OT and placebo administration in fear recognition. The independent variables were participants' performance following placebo administration as a measure of baseline ability and order of treatments (OT/placebo first). Given our hypothesis regarding a greater enhancement effect of IN OT among participants whose performance on the task was poor, the prediction tested in this analysis was that lower placebo performance would be significantly associated with greater drug effect, and the *p*-values would be one-tailed accordingly. The regression analysis showed that placebo performance predicted the size of the drug effect, *R* Square = 0.205, *p* < 0.001. The direction of the relationship was as predicted: enhancement effects were larger for individuals whose performance was lower. Thus, we divided the participants into two groups based on the median of their baseline (placebo) performance for fear recognition (median = 0.5): below-median performance and above-median performance. To investigate the difference between the two groups, we conducted separate paired *t*-tests (with Bonferonni corrections) for each group, comparing their fear-recognition performance following OT administration to that following placebo administration. As shown in Figure 2, a significant difference was evident only for the below-median group [*t*_(23)_ = 4.575, *p* = 0.0001], indicating that participants whose baseline performance was below the median performed significantly better following IN OT administration [Mean = 43.88 (*SD* = 25.29)] as compared to placebo [Mean = 23.58 (*SD* = 13.67)], Cohen's *d* = 1.056. The above-median performance group did not show a similar significant difference [*t*_(23)_ = −0.296 ns] between IN OT administration [Mean = 63.37 (*SD* = 21.47)] and placebo administration [Mean = 64.34 (*SD* = 12.41)]. In order to examine the possible moderating role of psychiatric status in the context of the described regression model, we performed a moderated regression, using the group factor (patients/control) as the moderating factor (mediator), according to Baron and Kenny (1986)'s model. No significant relationship was found between the regression independent variable (i.e., the performance of participants in fear recognition following placebo administration) and the mediator (*B* = −2.195, *p* > 0.05).

## Discussion

The aim of the current study was to characterize the effect of intranasal administration of OT on fear recognition in individuals with schizophrenia and in healthy controls. In line with our previous study (Fischer-Shofty et al., 2010), our results show that indeed participants were able to detect fearful facial expressions more accurately following IN OT administration regardless of their psychiatric status (e.g., healthy vs. patients). However, it is important to note that not all studies have found a specific improvement in the recognition of fear following IN OT administration, and rather reported a general effect on emotion recognition in patients with schizophrenia (Averbeck et al., 2012) and in healthy individuals (Schulze et al., 2011; Lischke et al., 2012a,b; Van and Bakermans-Kranenburg, 2012). Furthermore, in contrast to Marsh et al. (2010), the current results did not show any effect of IN OT administration on recognition of happiness. Notwithstanding the importance of these studies, they were all limited by relying primarily on still emotional faces. The task used in the present study is highly ecologically valid as it mimics the dynamics of gradual changes in facial expressions. Thus, it is possible that the difference between previous studies and the present study is derives from the use of different tasks of emotion recognition.

A second goal of this study was to investigate whether IN OT affects differently patients and controls. In contrast with our original hypothesis, IN OT did not have a differential effect on patients and controls. While IN OT had a general effect on improving fear recognition, it did not change performance in the groups separately. In the next stage we wanted to examine the differential impact of IN OT depending on individual baseline ability of fear recognition. Our results show that IN OT improved the performance of participants whose baseline performance levels in fear recognition was below the median, while significant difference between IN OT and placebo trials was evident in the above-median subgroup. These findings are in line with the work of Bartz et al. (2010), who reported a selective enhancing effect for intranasal administration of OT on empathic accuracy. In their study, participants were rated according to the Autism Spectrum Quotient (AQ) to determine their basic social capability. While the performance of the more capable subgroup (i.e., lower AQ scores) was not affected by IN OT administration, participants with high AQ scores performed the task better following OT administration. These findings by Bartz, et al. suggest a more “circumscribed” role for IN OT's augmentation of social salience, one that preferentially benefits those with lower baseline capabilities. As mentioned by Bartz et al., these results are in line with the proposition made by Shamay-Tsoory et al. (2009) that IN OT exerts its effect by enhancing the salience of social cues, thus increasing their importance as sensory input. Therefore, it may be that IN OT will mostly benefit those who have deficits in the perception of valuable social signals, as is the case in schizophrenia and other disorders.

Numerous studies have established the dominant and crucial involvement of the amygdala in emotion recognition and particularly in fear processing (see Adolphs, 2008). More recently, increasing evidence has linked OT to the amygdala, mainly as an effect of activation reduction (Kirsch et al., 2005; Pittman and Spencer, 2005; Domes et al., 2007a,b, 2010; Baumgartner et al., 2008; Riem et al., 2012; Rupp et al., 2012), thus associating OT and fear perception. In this regard, it is worth mentioning the study by Huber et al. (2005), which reported an excitatory effect for OT in a distinct part of the amygdala (central and capsular division of the central amygdala). Findings like these suggest a neuromodulatory role for OT in amygdala and brainstem circuits (see Stoop, 2012 for recent review) which may underlie OT's ability to increase the accurate perception and interpretation of survival-related social cues (i.e., facial expressions of fear) (Fischer-Shofty et al., 2010; Labuschagne et al., 2010; Lischke et al., 2012a,b). While OT seems to be useful in heightening one's attention to social cues (Shamay-Tsoory et al., 2009), which might be helpful for individuals who exhibit impaired social attention, it is also worth considering the ramifications of exaggerated social salience. These latter effects may be important in individuals with a maladaptive, hypervigilant social perception system, as can be seen in Borderline personality disorder (Fertuck et al., 2009; Bartz et al., 2011; Frick et al., 2012). Our results are linked to previous data indicating the beneficial effect of IN OT administration on social cognition in schizophrenia. They are also in line with previous studies that reported an association between endogenous levels of OT and social cognition in schizophrenia (Goldman et al., 2008; Rubin et al., 2010; Sasayama et al., 2012), those reporting links between OXTR genes and schizophrenia (Souza et al., 2010a,b; Montag et al., 2012a,b), as well as studies examining the effect of intranasal administration of OT on social cognition in schizophrenia as a single dosage (Averbeck et al., 2012; Goldman et al., 2011) or as a more long-term treatment (Feifel et al., 2010; Pedersen et al., 2011; Modabbernia et al., 2013). These findings further reinforce the hypothetical role of the oxytocinergic system in the epidemiology of this disorder and suggest OT as a possible therapeutic target in schizophrenia.

It is important to note that recent studies suggest a dissociable role for IN OT in males and females. Domes et al. (2010) reported a different neural activation pattern, including the amygdala, following IN OT administration in women, as opposed to men. Similarly, Lischke et al. (2012a,b) have found an increased activation response of women's amygdala following IN OT administration, as opposed to the known reduced activation of the amygdala in men (Kirsch et al., 2005). The sample in the current study included both men and women (with the majority of men), and while our reanalysis of the data using only male participants remained marginally-significant, future studies should use larger samples of women and men and take into consideration sex differences in the context IN OT.

The study has some limitations that should be taking into consideration. One relates to sample size and sample composition. The participants in the current study were healthy individuals and patients with schizophrenia, while other psychiatric populations were not represented in our cohort. Therefore, general implications regarding the effects of intranasal administration of OT on fear recognition should be made with caution. Moreover, our power analysis suggests that a larger sample (more than 150 participants) could provide significant results regarding the contribution of psychiatric status to the reported results. Thus, future studies may examine the study hypotheses using larger samples in order to provide the basis for stronger conclusions. Our results show a general effect of IN OT on fear recognition, while no significant effect was evident in either the patients group or in the healthy control group alone. Therefore it is impossible to infer whether the effect of oxytocin is driven by the patients or by the healthy controls. Moreover, as our regression model indicates, it cannot be concluded which of the groups (i.e., patients or healthy controls) is responsible for these results. In view of the fact that several psychiatric disorders show meaningful deficits in emotion recognition, future studies should further examine this possible effect in larger groups, as well as for other types of social inadequacy seen in mental illnesses such as anxiety, depression, and autism spectrum disorders. In regard to study cohort, future studies should address important issues in the context of schizophrenia, such as illness stage, medication type, and comorbidity, which could potentially affect the results. Finally, it should be noted that in the current study we used only two basic emotions, happiness, and fear. Therefore, any generalization of the effect of IN OT on emotion recognition, other than those who were evaluated in the study must be also done with caution. Moreover, it has been suggested that fearful facial expressions are better recognized in masked faces that lack the mouth region, as opposed to facial expressions of happiness. Thus, we cannot rule out the effect of the masked stimuli used in the current study, which could have had an effect on our results. Another aspect of the study design that could affect our results involves the use of performance following placebo administration as baseline performance. While the use of placebo trials has been reported in previous studies of different drugs and cognition (Farah et al., 2009), the design could be improved by having a second measure of performance following placebo to prevent the use of our predictor variable in calculating the drug effect. Moreover, differences between patients and healthy control participants in education level, as well as intelligence level as assessed by the Shipley test, may also serve as study limitations. Although our controlled analysis did not blemish the original result of interaction between treatment and emotion recognition, nonetheless, future studies which will control for these factors a priori could have stronger results. Therefore, we cannot confidently conclude that IN OT is solely responsible for the apparent difference in emotion recognition. Future studies should rule out the effect of education and intelligence levels on these abilities by employing a matched-group design regarding these factors.

### Conflict of interest statement

The authors declare that the research was conducted in the absence of any commercial or financial relationships that could be construed as a potential conflict of interest.
